# Rapid shape detection signals in area V4

**DOI:** 10.3389/fnins.2014.00294

**Published:** 2014-09-16

**Authors:** Katherine F. Weiner, Geoffrey M. Ghose

**Affiliations:** ^1^Graduate Program in Neuroscience, University of MinnesotaMinneapolis, MN, USA; ^2^Departments of Neuroscience, Psychology, and Radiology, Center for Magnetic Resonance Research, University of MinnesotaMinneapolis, MN, USA

**Keywords:** reaction time, visual decision making, ventral visual stream, rapid shape detection, foveation, saccades, temporal precision

## Abstract

Vision in foveate animals is an active process that requires rapid and constant decision-making. For example, when a new object appears in the visual field, we can quickly decide to inspect it by directing our eyes to the object's location. We studied the contribution of primate area V4 to these types of rapid foveation decisions. Animals performed a reaction time task that required them to report when any shape appeared within a peripherally-located noisy stimulus by making a saccade to the stimulus location. We found that about half of the randomly sampled V4 neurons not only rapidly and precisely represented the appearance of this shape, but they were also predictive of the animal's saccades. A neuron's ability to predict the animal's saccades was not related to the specificity with which the cell represented a single type of shape but rather to its ability to signal whether any shape was present. This relationship between sensory sensitivity and behavioral predictiveness was not due to global effects such as alertness, as it was equally likely to be observed for cells with increases and decreases in firing rate. Careful analysis of the timescales of reliability in these neurons implies that they reflect both feedforward and feedback shape detecting processes. In approximately 7% of our recorded sample, individual neurons were able to predict both the delay and precision of the animal's shape detection performance. This suggests that a subset of V4 neurons may have been directly and causally contributing to task performance and that area V4 likely plays a critical role in guiding rapid, form-based foveation decisions.

## 1. Introduction

Humans and other primates explore their visual world through rapid, serial fixations lasting only several hundred milliseconds (Einhäuser et al., 2006). In these brief fixations, extrafoveal visual representations must be used to select the next saccadic target location based on salience or behavioral importance. However, the neural basis of these foveation decisions is unclear. A particular challenge is that neurons contributing to these decisions must not only be able to signal the appearance of salient objects or shapes within hundreds of milliseconds, but that signal must be read-out by oculomotor neurons with similar temporal precision in order to direct the upcoming saccade.

We hypothesized that neurons in area V4 may provide the precise and reliable signals necessary for such foveation decisions. Neurons in area V4 representing extrafoveal visual space are known to respond to contour features and shapes defined by cues including luminance contrast (Pasupathy and Connor, 1999), chromatic contrast (Bushnell et al., 2011), and motion (Mysore et al., 2008; Handa et al., 2010). Additionally, recent studies of the representation of figure/ground (Poort et al., 2012), illusory contours (Pan et al., 2012; Cox et al., 2013), and the integration of contour elements (Chen et al., 2014) suggest that area V4 may play a vital role in object detection by using visual cues to group elements of objects together and segment them from their surroundings. These stimulus driven responses can be very rapid (60–120 ms), and therefore are potentially well suited for rapid foveation decisions.

Neurons in area V4 also project to areas involved in the generation of attentional and saccadic signals, such as prefrontal and parietal cortex (FEF and LIP, respectively; Ungerleider et al., 2008) and the superior colliculus (Gattass et al., 2013), suggesting that object detection in area V4 could result in the direction of attention or saccades to the object location. There is also electrophysiological evidence to suggest that area V4 is an important contributor to visually-based behavior. These neurons strongly modulate their sensory responses according to behavioral relevance (Chelazzi et al., 2001; Ogawa and Komatsu, 2006; Mirabella et al., 2007; Ipata et al., 2012) and may contribute to visual working memory (Liebe et al., 2012; Hayden and Gallant, 2013). Moreover, several studies have attempted to link trial-to-trial variations in stimulus response with performance of various tasks: feature-specific responses to color or orientation (Mirabella et al., 2007), coarse noisy orientation discrimination (Zivari Adab and Vogels, 2011), and disparity discrimination (Shiozaki et al., 2012).

While these studies suggest that V4 neurons may carry both stimulus- and choice-related signals that could play a central role in foveation decisions, they have not examined the moment-to-moment reliability of both of these types of signals simultaneously in the context of a rapid decision. To address whether V4 responses reliably reflect the presence of shapes and predicted subsequent saccades over timescales necessitated by the frequency of saccades in natural vision, we recorded from populations of V4 neurons while monkeys performed a rapid shape detection task in which they were required to foveate upon a briefly presented shape embedded in noise. We found that many V4 neurons were able to significantly signal when a shape appeared and/or predict the animal's behavior on a moment-by-moment basis within the timeframe of the animals' reaction times. The majority of these cells were unlikely to contribute to detection decisions in a causal, feedforward manner because activity related to the stimulus and animal's behavior either did not overlap in space and time (Choe et al., 2014) or was not precise enough to explain behavior (Parker and Newsome, 1998). However, the activity from a fraction of neurons was consistent with both behavioral precision and delay. These results suggest that area V4 is intimately involved in decisions to saccade to visual stimuli, with many neurons modulated by saccadic preparation or behavioral relevance and a few neurons potentially contributing directly to rapid shape detection decisions.

## 2. Materials and methods

### 2.1. Ethics statement and surgical procedures

All procedures involving animals conformed to guidelines established by the National Institutes of Health and were approved by the Institutional Animal Care and Use Committee of the University of Minnesota. Animals were initially anesthetized with ketamine and anesthesia was maintained with isoflurane throughout all surgical procedures. Analgesics and antibiotics were administered during and following all surgeries to minimize discomfort and prevent infection. To stabilize head position during training and recording sessions, headposts (titanium or PEEK polymer) were chronically implanted under sterile surgical conditions. Animals were fully acclimated to their primate chair and training room before headposts were used for stabilization. Once each animal was trained on the shape detection task, a microelectrode array (Blackrock Microsystems) was chronically implanted, again under sterile conditions.

### 2.2. Task

We trained two experimentally naïve male monkeys (*Macaca mulatta*, ≈7 and 13 kg) in a challenging shape detection task. While the animals were performing the task, head position was stabilized by a chronically implanted headpost and eye position was monitored by an infrared eye tracker (Arrington Research). Each trial began with the appearance of a fixation dot. After ≈500 ms of fixation, a noise stimulus appeared at a peripheral location. The animals were required to maintain fixation until an enclosed shape was briefly presented in a background of noise. Both shape identity and timing of presentation were randomly determined for each trial. Presentation times were drawn from an exponential distribution, with means set 500 ms for Monkey Z and 1000 ms for Monkey J. Because false alarms were frequent, the mean of this distribution ended up slightly shifted toward earlier times (actual mean time to shape appearance was Monkey Z: 460 ms and Monkey J: 970 ms).

The distribution of shape appearance, and the fact that shapes were only briefly presented (Monkey Z: 83 ms and Monkey J: 120 ms), encouraged the animals to maintain a high level of vigilance throughout the trials (Ghose, 2006). Animals were required to signal their awareness of shape appearance by making an eye movement to the shape within a reaction time window (150–550 ms) to receive a juice reward. If the animals failed to make a saccade within this window, the trial ended without reward. Trials also ended without reward if the animal broke fixation before a shape appeared. In ≈5% of trials, no shape appeared, and the animals were rewarded for maintaing fixation throughout the length of the trial. During initial training of the animals, the noisy background in which the shape was embedded was at low contrast, but as training progressed, the contrast of a surrounding noise stimulus was gradually increased. At the end of training, and during all recording sessions, elements of the noise and shape stimuli appeared at the same contrast. This ensured that no low-level cues were associated with shape appearance.

### 2.3. Visual stimulation

Visual stimuli were delivered on an LCD monitor (120 Hz). A photodiode affixed to the screen confirmed the timing of stimulus presentation. The stimulus consisted of a 7 × 7 array of achromatic Gabors. The stimulus array was positioned to overlap with the receptive fields of recorded cells; it was centered at an eccentricity of 3.75° (azimuth: 3.75°, elevation: 0.2°) for Monkey Z and an eccentricity of 5.5° (azimuth: − 2.5°, elevation: -4°) for monkey J. In both animals, the radius of each Gabor element was 0.38°, resulting in receptive fields containing ~16–25 elements (Gattass et al., 1988; Motter, 2009). The spatial frequency was 2°/cycle.

The orientation of each Gabor in the array was randomly and independently set to one of eight different values to create noise. To eliminate motion cues as a potential confound for contour detection, the noise stimulus was constructed by interleaving two types of these noise frames among frame updates: static and redrawn. A single static noise frame was generated at the beginning of each trial, but was not varied within a trial, such that the pattern was consistent between successive presentations. In contrast, a new random pattern was generated for each redrawn noise frame, such that pattern varied between successive presentations. Our framerate of 120 Hz meant that each static/redrawn frame was present for ≈8 ms. During shape presentation, the Gabors defining the shape replaced the corresponding Gabors within the static noise frame, but this combined static-shape frame continued to be interleaved with redrawn noise frames.

The shapes to be detected were defined by fixing the orientations of 16–19 adjoining Gabor patches so as to form a contiguous contour. During recording sessions, the Gabor elements of both shapes and noise appeared at the same contrast (45–50%). Three different shape stimuli were used. Monkey Z was taught to report the presence of any of these shapes at four different orientations (for a total of 12 shape stimuli). Because he tended to work for fewer trials, only one orientation of each shape was presented to Monkey J.

### 2.4. Electrophysiology

Once the animals were trained to perform the task in the absence of any contrast differences between shape and background noise, a 10 × 10 microelectrode array (1 mm length, injected with a 1 mm pneumatic inserter; Blackrock Microsystems) was chronically implanted in visual area V4 on the prelunate gyrus (Monkey Z: left hemisphere, Monkey J: right hemisphere), slightly above the tip of the inferior occipital sulcus. Spike times and waveforms were recorded as the animals performed the task and then sorted offline using the Waveclus toolbox (Quiroga et al., 2004). Data from 9 sessions in Monkey Z and 11 in Monkey J were initially considered. Each of these sessions had at least 375 trials in which the monkey maintained fixation until a saccade was made to the stimulus location or the trial ended. Over these 20 sessions, 683 single- and multi-units were identified through spike sorting and met the minimum signal to noise ratio criterion of 2.2. No differences between single- and multi-units were ever observed, so they are presented together in the analyses. We further required cells to be visually responsive, with increased firing rates in response to the appearance of the noise stimulus. Specifically, the units were required to have a statistically (Wilcoxon signed-rank test, *p* < 0.05) larger response in the first 50–250 ms following noise stimulus onset than the preceding 200 ms. This left us with 464 units. Because the same unit often appeared to be present on a particular electrode across multiple recording sessions, analyzing all available data would have resulted in these stable cells being over-represented in our sample. To avoid this, we chose to use units from each electrode only once. For each electrode with cells in multiple recording sessions, we used only the data from the session with the greatest number of trials. The results presented here therefore include data from 8 recording sessions with Monkey Z and 10 sessions with Monkey J, with a total of 178 units.

### 2.5. Average event-aligned responses

To examine potential differences between trials in which a shape appeared and was detected, vs. trials when a shape appeared and was not detected, we analyzed firing rates of individual units during the first 225 ms following shape appearance. Trials with a saccade during this period of time were excluded so that all included trials had reaction times of at least 225 ms. To examine potential differences between trials in which the animals correctly reported the presence of a shape and those in which the animal made a saccade when a shape had not yet appeared, we analyzed firing rates of individual units in the last 225 ms preceding the saccade, excluding trials with reaction times shorter than 225 ms. In both the post-shape and pre-saccade analyses, spikes were counted within a 50 ms bin moving in 10 ms steps and then averaged across trials.

### 2.6. Mutual information conveyed by single cells

Each trial contained three types of simultaneously observed variables: the noise/shape history (stimulus), neuronal discharge of the multiple units sampled by the microelectrode array (neuronal activity), and eye position (behavior). We used a mutual information analysis to quantify the reduction in uncertainty about one task variable given knowledge of another task variable, on a moment-by-moment basis. This method has been described in detail in previous publications Ghose and Harrison (2009); Harrison et al. (2013).

Briefly, visual stimulus and behavioral response variables were treated as binary point processes (shape/noise, saccade/fixation), with “shape” occurring at shape onset and “saccade” at fixation window exit, respectively. The neuronal activity variable was quantified as the number of a unit's spikes. The uncertainty of each of these variables is quantified by entropy *H*

(1) $$H_{x}=-\sum_{x}p_{x}\log(p_{x})$$

where *p_x_* is the probability of observing the the variable at value *x*.

The reliability of the relationship between pairs of variables was quantified in units of bits, using the direct method of mutual information calculation. Behavioral reliability was quantified as the mutual information between stimulus and subsequent behavior (*I_behav_*). Sensory reliability was quantified as the mutual information between stimulus and subsequent spike count (*I_sensory_*), and choice reliability was quantified as the mutual information between spike count and subsequent behavior (*I_choice_*).

(2) $$I_{behav}=\;H_{stim}+H_{eye}-H_{stim,eye}$$

(3) $$I_{sensory}=H_{stim}+H_{activity}-H_{stim,activity}$$

(4) $$I_{choice}\;=\;H_{eye}+H_{activity}-H_{eye,activity}$$

where *H_x,y_* is the joint entropy between the variables.

To avoid assumptions regarding timing and homogeneity of neuronal responses, mutual information was calculated at a range of binwidths (multiples of 25 from 25–250 ms) and delays (multiples of 5 from 0–500 ms for behavioral information and 0–250 ms for sensory and choice information). Plotting the mutual information at each combination of delay and binwidth results in an information surface that depicts how reliability varies as different temporal parameters are considered. Because the average reaction time of both animals was less than 250 ms and we did not include data occurring after a saccade, larger binwidths became very poorly sampled. For behavior we included delays up to 500 ms, so that the information “peak” could be seen to fall off in all directions. We limited sensory and choice delays to 250 ms, because, given the short reaction times, longer days would not have been behaviorally relevant.

All trials in which the animals acquired fixation and the stimulus appeared were included in this analysis. In the case of sensory and choice reliability, this resulted in the same data contributing to each surface, including neuronal responses. Differences in sensory and choice reliability therefore directly reflect differences in the strength of the relationship of these responses with either the stimulus or animal's behavior, respectively. Additionally, including all available data allowed for the best estimate possible of each cell's response properties and therefore more accurate information estimates. Trial events and spiking activity were included from 60 ms after noise stimulus onset until either a saccade was made or the trial ended. Including activity prior to this would potentially have resulted in artificially decreasing the firing rates observed in response to the noise condition; however, if the initial 60 ms of stimulus onset were included, the results changed very little.

For a given combination of delay and resolution, each trial was divided into bins of the appropriate width, aligned to shape onset for behavioral and sensory information, or to saccade onset for choice information. This alignment was different than previously used in Ghose and Harrison (2009) and Harrison et al. (2013) but ensured that a delay at a given binwidth always represented a consistent period of time relative to shape onset or fixation offset. Results were largely unchanged if different alignments were used. For each type of information, a contingency table was updated according to the states of the two variables of interest. Once all trials had been parceled in this manner, the contingency table represented the relationship between the two variables at the given binwidth and separation and was used to calculate the entropies required by Equations 2–4. Dividing the mutual information (bits) by the binwidth converted this value into mutual information rate (bits/s).

Because mutual information has an inherent positive bias (Treves and Panzeri, 1995), we corrected for the information rate that would be expected by chance if there was no relationship between the variables. To calculate expected chance information rates, the contingency tables used to calculate mutual information at each delay and binwidth were all resampled 100 times. Preliminary analyses showed that bias estimates were extremely similar if tables were resampled 1000 times. For sensory and choice tables, the number of observations for each stimulus or behavioral condition was held constant, and spike counts were sampled based on the probability of occurrence across conditions of the variable. This tends to maintain the probability of observations in any one variable, but destroys the relationship between variables. Values that were not deemed to be “significant” (above the 95th-highest bootstrap value) were set to zero. The average bootstrap value was subtracted from significant values.

The contingency tables can also be used to address covariances among the three variables (Ghose and Harrison, 2009; Harrison et al., 2013). Correcting for these covariances ensures that our sensory information computations were not simply the result of covariance between choice-related neuronal activity and an animal's behavior, or conversely, that choice information was not the result of covariance between stimulus-related activity and behavior. The covariance correction consists of using the probabilities described in two contingency tables (for example, sensory and behavior), to generate a third chance contingency table (in this case, choice).

If *p_shape_*(*n*) = *p*[*activity* = *n*|*stim* = *shape*](*d*_1_) describes the probability of observing *n* spikes at a delay *d*_1_ after the appearance of a shape, and *p_shape_*(*saccade*) = *p*[*eye* = *saccade*|*stim* = *shape*](*d*_2_) is the probability of observing a saccade at a delay *d*_2_ after the appearance of shape, then the probability of observing *n* spikes at delay *d* = *d*_2_ − *d*_1_ prior to the saccade, solely due to these relationships with the shape, is the product of *p_shape_*(*n*) and *p_shape_*(*saccade*). The total probability of observing *n* spikes at delay *d* prior to the saccade is found by summing the probability given a shape (as described) with the probability given the noise stimulus. This total probability can then be used to update the appropriate location (corresponding to *saccade, n*) in the chance contingency table.

At a given binwidth, a chance choice delay (80 ms for example) could result from many different combinations of sensory and behavior delays at that binwidth (sensory delay: 120 ms, behavior delay: 200 ms; sensory 125 ms, behavior 205 ms; sensory 130 ms, behavior 210 ms; etc.). A predicted choice contingency table is therefore created for each of these possible combinations and used to compute mutual information. The maximum predicted mutual information is then subtracted from the observed choice information at that delay and binwidth to correct for covariance.

Because the mutual information is computed over a range of binwidths and delays, a single surface has a large number of points. For example, with 500 points on a surface (10 binwidths × 50 delays), we would expect approximately 25 points on this surface to fall above the 95th percentile of the resampled values by chance. When representing the reliability of a unit by the peak, or maximum, value on its information surface, we therefore imposed a false alarm criterion to correct for multiple comparisons. If the number of observed significant points did not exceed the number of significant points expected by chance, a unit's surface was considered to be flat with a non-significant peak. This likely resulted in an underestimation of the number of units that were informative about either the sensory stimulus or the animal's choice; however, because the same criteria were applied to both types of information in all cells, we could make relative comparisons. By examining peak magnitudes resulting from different sized subsets of data, we determined that recording sessions needed to include at least 375 usable trials (where the animal maintained fixation until either the trial ended or he saccaded to the stimulus patch) to obtain consistent estimates.

### 2.7. Specificity of shape responses

Using a two-way analysis of variance, we determined the extent to which a unit's response to shape appearance was influenced by the identity of the shape. Shape responses in the 75–200 ms following shape appearance and noise responses in the 125 ms preceding that period were analyzed. All trials in which a shape appeared and a saccade did not occur within the analysis window were included. Results were indistinguishable if only correct trials were analyzed. The factors considered in the two-way analysis of variance were stimulus and shape identity. The stimulus factor had levels of noise and shape. The shape identity factor had three levels for Monkey J and 12 levels for Monkey Z (3 shapes at 4 orientations each). The interaction term thus tests whether stimulus responses are the same across the different shape identities. The percent of explainable variance due to this interaction was determined for each unit by dividing the Mean Squares of the interaction by the sum of the Mean Squares from the interaction, the shape/noise factor, and the shape IDs. Partial correlations among this measure of shape specificity, sensory information, and choice information, were computed across units with non-zero sensory and choice information (94 out of 171 units).

### 2.8. Predictions of behavior

We used each unit's sensory and choice surfaces to generate surface-based predicted behavior. This is the behavior that would arise, given the statistical relationship of a neuron to stimulus and behavior, if behavior was completely derived from that a neuron. This is a simple feedforward model in which a stimulus evoked response drives behavior. In such a model, there are many sensory and choice delays, at a given binwidth, that can sum to result in the same behavior delay. Thus, for each behavior delay, we computed the product of sensory and choice information (in bits) for every possible delay combination. The maximum product was taken as the behavior prediction for the delay and binwidth of interest and converted to information rate (in bits/s). This process is similar, but somewhat simpler than, the contingency table-based behavior predictions previously produced by Ghose and Harrison (2009) and Harrison et al. (2013). The contingency table-based method locks the response categories such that a sensory response of 5 spikes can only be combined with a choice response of 5 spikes. With the current data set, the contingency table-based method gave very similar results to those of the surface-based predictions presented here, but the table-based method requires more data to generate a smoothly shaped surface.

Overlap between predicted and observed behavior surfaces was calculated in the binwidth (125 ms) associated with the peak information rate in the observed average behavior surface. The average behavior surface was used as a reference because there was very little variation in this surface between animals or across days. To calculate overlap between a predicted surface and observed surface, information at all delays for a binwidth of 125 ms was normalized to the peak information rate of that binwidth. The crossproduct of these two normalized delay plots was considered to be the overlap.

### 2.9. Hardware and software

Behavioral control and visual stimulation were computer controlled using customized software (http://www.ghoselab.cmrr.umn.edu/software.html). Electrophysiological data were acquired via a Blackrock Microsystems Neural Signal Processor, using a combination of their Central software and customized software. All data were converted to MATLAB format using the Neural Processing MATLAB Kit (NPMK, Blackrock Microsystems). Analysis was mainly performed with custom MATLAB software. Hartigan's dip test was performed with HartigansDipSignifTest.m by F. Melcher.

## 3. Results

**Figure 1:**
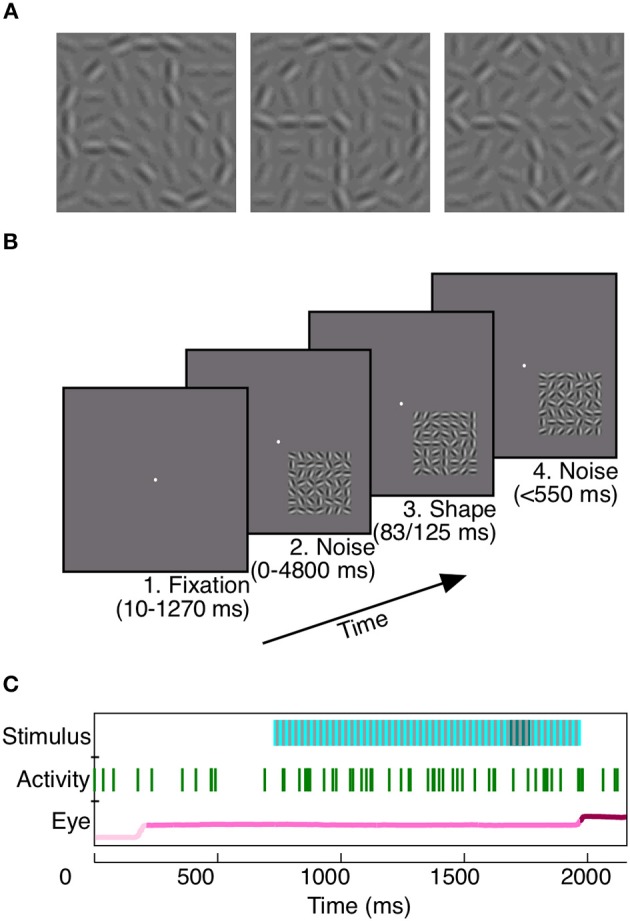
**Rapid shape detection task**. Each monkey was trained to detect the appearance of any of three shapes **(A)**. For Monkey Z, each of the three shapes could appear at any one of four orientations, for a total of 12 potential shape frames. In **(A)**, shapes are shown at a higher contrast than background for the purpose of illustration; noise and shape elements were at equal contrasts during recording. The task was to remain fixated on a centrally located point **(B1)** during noise stimulus presentation **(B2)**. When a shape briefly appeared, embedded in the noise stimulus (**B3**, same shape as **A** middle), the animals were required to make a saccade to the location of the stimulus within 150–550 ms **(B4)** to receive a juice reward. Our analyses rely on three simultaneously recorded variables: the presented stimulus, neuronal activity, and eye position **(C)**. To prevent the animals from using motion cues for shape detection, static noise frames (light blue, **C**, Stimulus), in which the random noise pattern did not vary on successive presentations, were interleaved with redrawn noise frames (gray, **C**, Stimulus). When a shape appeared (dark blue, **C**, Stimulus), it was embedded in the static noise frames. Green vertical lines indicate spikes recorded during the trial from an example unit. The eye position in light pink prior to fixation, pink during fixation, and dark pink subsequent to the saccade.

To study the potential contribution of area V4 to rapid shape detection, we first trained two monkeys to detect a shape which was briefly presented (Monkey Z: 83 ms, Monkey J: 125 ms) at a random time within a background of dynamic noise (Figure 1). At each moment in the trial, the monkeys had to decide whether a shape was present. This task design, as well as the brevity of shape presentation, encouraged consistent vigilance from the animals.

**Figure 2:**
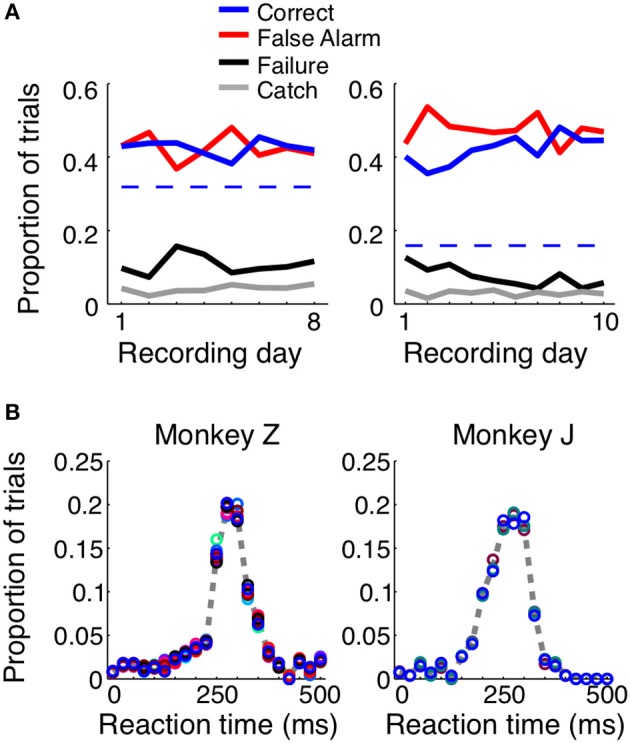
**Trial outcomes across days and reaction times for each animal**. Both animals correctly detected the presence of a shape ~40–50% of the time (**A**, solid blue lines). Dashed blue lines represent the percent correct that could be achieved by random guessing. False alarm trials, in which the animal incorrectly reported the presence of a shape before one appeared, were equally prevalent (red lines). The animals failed to respond to the presence of a shape ~10–20% of the time (black lines), and in ~5% of the trials, the animals remained fixated throughout the duration of a catch trial, when no shape appeared. The reaction time distributions were similar for both animals **(B)** and across shapes (represented by circle color). If the animals were randomly guessing, these distributions would be flat.

When consistently working, the monkeys correctly reported the presence of a shape in ~40% of the trials, with average reaction times of 248 ms for Monkey Z and 237 ms for Monkey J (Figure 2). For each animal we used the total length of trials, the size of the reaction time window (400 ms), and the number of total number of shape appearances to calculate the percent of correct detections that would result from blind guessing (total time in which responses would be considered correct/total time a stimulus was present). In Monkey Z this chance level was 32% and in Monkey J it was 16% correct. The chance level is lower in Monkey J because the length of his trials were intentionally longer; however, both monkeys perform above chance. Additionally, if the monkeys were blindly guessing, as opposed to actually detecting the appearance of the shape, we would expect the reaction times to be evenly distributed, and this is obviously not the case (Figure 2B). When the animals made correct decisions, they did so with temporal precision: most reaction times occurred within a 100 ms window centered around the mean. Most incorrect trials resulted from an early response (i.e., a false alarm), while the animals failed to detect the appearance of a shape ~10% of the time.

While the animals performed the task, we recorded neuronal activity with a microelectrode array chronically implanted in area V4. The stimulus was positioned to achieve maximal response from neurons whose activity was recorded by the array, and the size of the stimulus was chosen so that each neuron's receptive field would contain 15–25 elements of the stimulus array (Gattass et al., 1988; Motter, 2009). The data presented here comes from 8 recording sessions in Monkey Z and 10 sessions in Monkey J and includes 178 units (see Materials and Methods for inclusion criteria).

### 3.1. Average event-aligned responses

**Figure 3:**
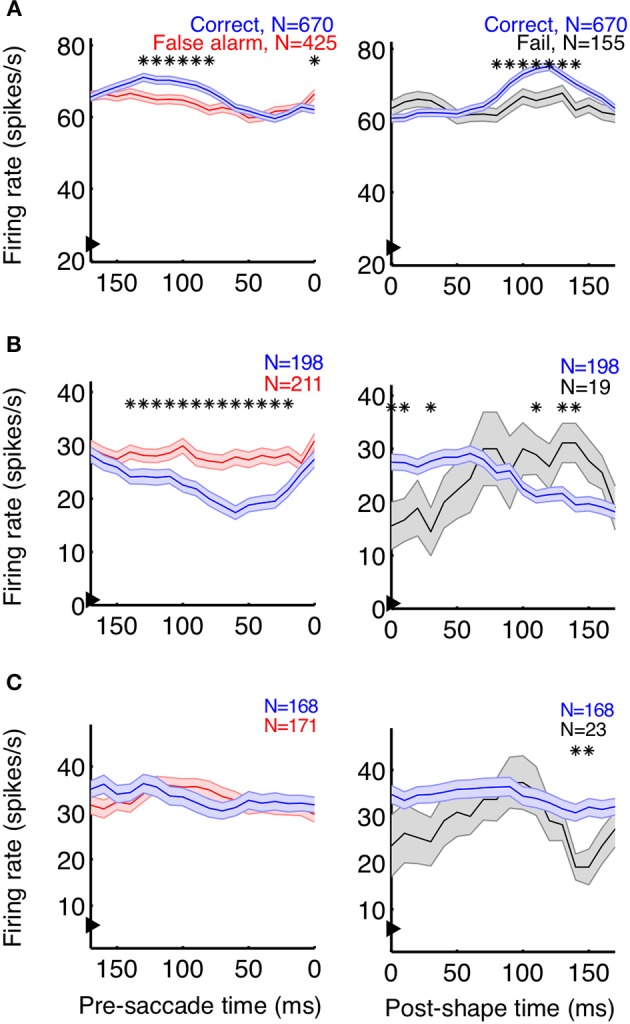
**Event-aligned firing rates of some units suggest task relevance**. Example units from Monkey Z **(A)** and Monkey J **(B,C)** are shown. The left plots depict the average saccade-aligned firing rates for correct (shape + saccade; blue) and false alarm (no shape + saccade trials; red) trials, demonstrating changes in firing rate due to the stimulus, when behavior is constant. The right plots depict shape-aligned firing rates for correct (shape + saccade; blue) and failure (shape + no saccade; black) trials, demonstrating changes in firing rate reflective of subsequent behavior when the stimulus is constant. The units in **(A,B)** appeared to reflect both the presence of a shape and the animal's detection of shapes, although firing rates were modulated in opposite directions and the effect in **(B)** between correct and failed trials was less clear. The neuron represented in **(C)** showed no clear task-relevant modulations. Firing rates were calculated in 50 ms bins, with the x-axis representing the bin edge closest to the relevant event (shape or saccade). Arrows on the y-axis indicate average baseline firing rate, from 150 ms before noise stimulus onset until 50 ms after. Shaded regions represent SEM, and asterisks indicate bins in which the firing rates of the two types of trials were significantly different (*p* < 0.05).

Neurons playing a pivotal role in shape detections should both signal the appearance of a shape and predict the animal's choices. As a first step to determine whether individual V4 neurons carry such signals, we plotted average event-aligned responses for individual units, separated by trial outcome. In both correct (shape + saccade; blue) and false alarm (no shape + saccade; red) trials, the animal reported the appearance of a shape (Figure 3, left column). Because the behavioral outcome was consistent between these trials, changes in saccade-aligned average firing rate could be at least partially attributed to differential responses to the sensory stimulus. Conversely, to examine the average change in firing rate attributable to the animal's behavior, we compared trials in which the stimulus was consistent (Figure 3, right column; correct: shape + saccade; blue, fail: shape + no saccade; black) and aligned these trials to shape onset.

In accordance with a role in shape detection, we did find neurons whose stimulus-aligned discharge was modulated by detection and whose saccade-aligned discharge was modulated by the stimulus. Interestingly, both types of modulation could occur in either the positive or negative direction (Figures 3A,B). We also found neurons with very little average modulation (Figure 3C). Although these results are suggestive that sub-populations of V4 neurons might participate in rapid shape detections, these analyses describe changes in the average firing rate of neurons over multiple trials within a recording session. By contrast, the animals' behavior during task performance must be based on changes in firing rate occurring on a moment-by-moment basis within a single trial. Additionally, much of our data were recorded in the presence of the noise stimulus, when the animals were fixating and thus indicating that they had not detected the presence of a shape. Traditional average firing rate analyses like those in Figure 3 ignore this period of decision-making (correct rejections), and our ability to discriminate choice modulations (for example in Figure 3B, between correct and failed trials) using only shape presentations is limited by the small number of failed trials. Finally, while the average firing rates suggested at least some of our neurons were modulated by stimulus and/or behavioral parameters, it is difficult to quantify and compare the strength of the relationship between the neuron's firing rates and these variables.

### 3.2. Task-relevant reliability of V4 neurons

**Figure 4:**
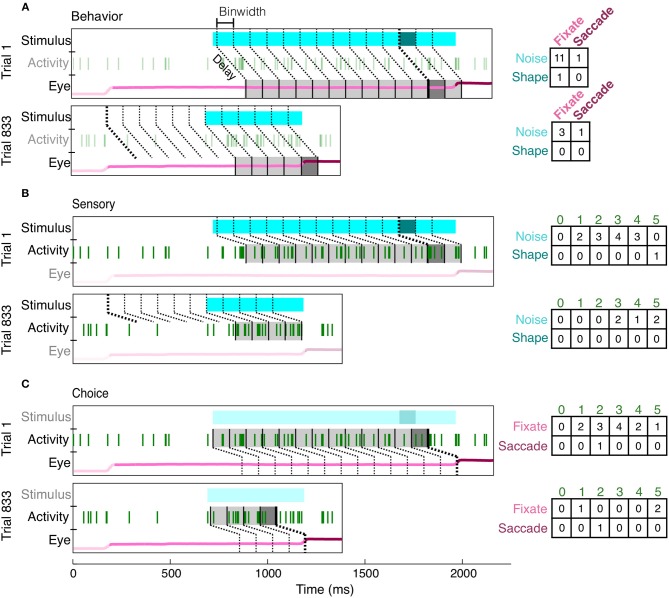
**Creation of contingency tables for behavior, sensory, and choice MI at a single delay (shown: 150 ms) and resolution (shown: 100 ms)**. For each type of MI, the same two example trials (Trial 1: correct, Trial 833: false alarm) are parsed differently, according to the two appropriate variables of interest (the third irrelevant variable has been dimmed in each subfigure). For behavior MI **(A)**, the two variables of interest are the stimulus (noise, light blue; shape, dark blue) and eye position (fixate, light pink; saccade, dark pink). Gray bins indicate how the eye position has been parceled according to the delay from the stimulus and the binwidth. The darkness of the bin (light or dark gray) indicates whether the stimulus at this delay was a shape or noise and therefore indicates which row of the contingency table (right) should be updated. Whether the animal was fixating or made a saccade within each bin determines which column is updated. The same conventions are used to show the parcellation for sensory MI **(B)**, in which the updated row is determined by the sensory stimulus at the given delay (indicated by darkness of gray bins) and the updated column is determined by the spike count (number of vertical green lines). For choice MI **(C)**, the contingency table is updated according to eye position and spike count. Bin edges were first aligned to either shape onset **(A,B)** or saccade onset **(C)** and then the appropriate delay was established. If the relevant alignment event did not occur, bins were aligned to fixation onset. Thicker bin edges indicate this point of alignment. When the contingency tables for each pair of variables are summed across all trials, they described the independent, conditional, and joint probabilities for each pair of variables. These probabilities were used to calculate MI and correct for covariance between the three variables.

To overcome many of the limitations of an average event-aligned analysis, we employed a mutual information analysis based on parceled trial data (Figure 4). This analysis enables us to quantify the reliability and temporal precision of the relationship between neuronal activity and task-relevant events on a moment-by-moment basis. Because the animals had to decide throughout the course of the trial whether or not a shape was present, and whether or not they should make a saccade, we wished to ask the same thing of our neurons. Essentially, how well would observing the activity of a neuron, at any point in the trial, improve one's chance of determining whether a stimulus had previously appeared, or if the animal was about to make a saccade? Our analysis therefore includes all data recorded in the presence of the noise or shape stimulus. Finally, because MI quantifies only the strength of the relationship between two variables, consistent increases or decreases in firing rate are quantified in the same manner, and the reliability of their relationship with task events can be directly compared.

Mutual information (MI) measures the reduction in uncertainty about one variable, given knowledge of another variable. The MI between a neuron's firing rate and the sensory stimulus quantifies how reliably a neuron's firing rate indicates whether or not a shape was present. Likewise, to quantify how reliably each neuron predicted the decision to either maintain fixation or saccade, we calculated the MI between the animal's behavior (saccade/no saccade) and each neuron's firing rate. We also quantify behavior reliability as the MI between the sensory stimulus and the animal's choice.

**Figure 5:**
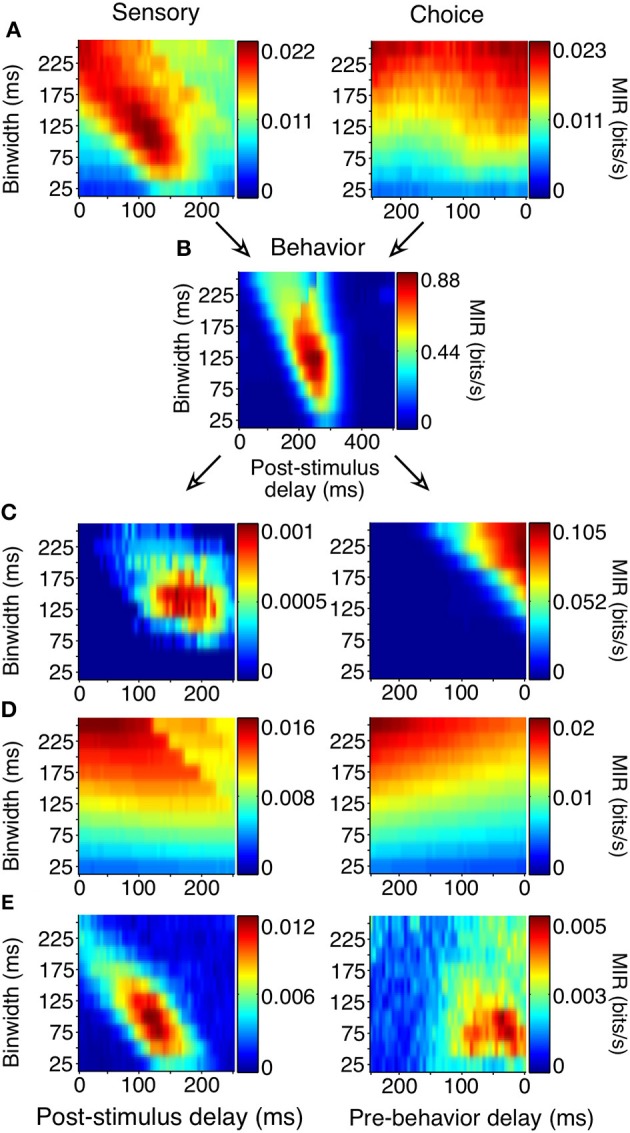
**Generation of sensory and choice surfaces**. For each combination of delay and binwidth, the relationship between the two variables of interest was tabulated across all trials (as in Figure 4). Each of these contingency tables was used to calculate the MIR, and a single corresponding point on the surface was colored accordingly **(A,B)**. The sensory information surface predicted solely by the covariance of choice information with the animal's behavior, and the choice surface predicted based solely by the covariance of sensory information with the animal's behavior were computed so that covariance could be accounted for **(C)**. Because each point on the surface was potentially based on a different number of observations, an estimate of bias was also obtained for each delay and binwidth through a bootstrapping procedure **(D)**. Due to the limited length of stimulus presentation and short reaction times, sampling decreased as binwidth and delay increased; this decrease in sampling increased the bias. The final surfaces **(E)** represent the information remaining after the covariance and bias for each point had been subtracted. All surfaces shown here represent the average across all 178 units.

To avoid making assumptions or generalizations about the time periods over which task-related relationships are most reliable, we computed MI using different delays (multiples of 5 from 0 to 250 for sensory/choice and 0 to 500 ms for behavior) and binwidths (multiples of 25 from 25 to 250 ms) to define an MI surface (Figure 5). All trials in which the animal acquired fixation and a noise stimulus appeared were analyzed, regardless of whether he made a saccade or not or whether a shape appeared or not. On the average surfaces, as well as many surfaces of individual neurons, there existed a single “peak,” or a combination of delay and binwidth over which information transmission was maximized. To ease comparisons of reliability between cells with MI peaks at different binwidths, all MI values (bits) were converted to Mutual Information Rate (bits/s, MIR). For behavior surfaces, the delay of the peak indicates the time at which there is the strongest relationship between the stimulus and the animal's response (similar to reaction time). The width of the bin containing the peak indicates the precision of his behavior (analogous to the width of the reaction time distribution). Similarly, for sensory surfaces, the delay of the peak indicates the time at which there is the strongest relationship between neuronal discharge and the preceding stimulus. On choice surfaces, the delay of the peak indicates the time at which there is the strongest relationship between neuronal discharge and the animal's subsequent response. For both sensory and choice surfaces, the bin width represents the neuron's precision: the length of time over which the neuron's firing rates must be considered to maximize MIR.

Correct performance of the animals' task required covariances between the animals' behavior and the stimulus. If they were behaving perfectly, they would always remain fixated during the noise stimulus and would only make saccades when a shape appeared. Such covariance can limit the ability to distinguish sensory and choice reliability. For example, if a neuron's firing rate always increased 150 ms before a saccade, and the animal's saccades precisely followed the appearance of shapes by 400 ms, there would be an increase in sensory information 250 ms following the appearance of a shape, even in a neuron whose firing rate was only modulated by the animal's behavior. Because of the relatively high false alarm rate, such covariances were not strong but nevertheless required consideration.

We accounted for the covariances by predicting the sensory surface based on the neuron's choice dependencies and the animal's behavior and by predicting the choice surface based on combining a neuron's sensory dependencies with the animal's behavior (Figure 5C, see Materials and Methods for detailed explanation). These covariance-predicted surfaces were then subtracted from the actual surfaces. Finally, because MI has an inherent positive bias (Treves and Panzeri, 1995), we corrected for that expected by chance, if there was no relationship between the variables (Figure 5D). Figure 5E shows the sensory and choice surfaces that result from this process, averaged across all recorded neurons.

**Figure 6:**
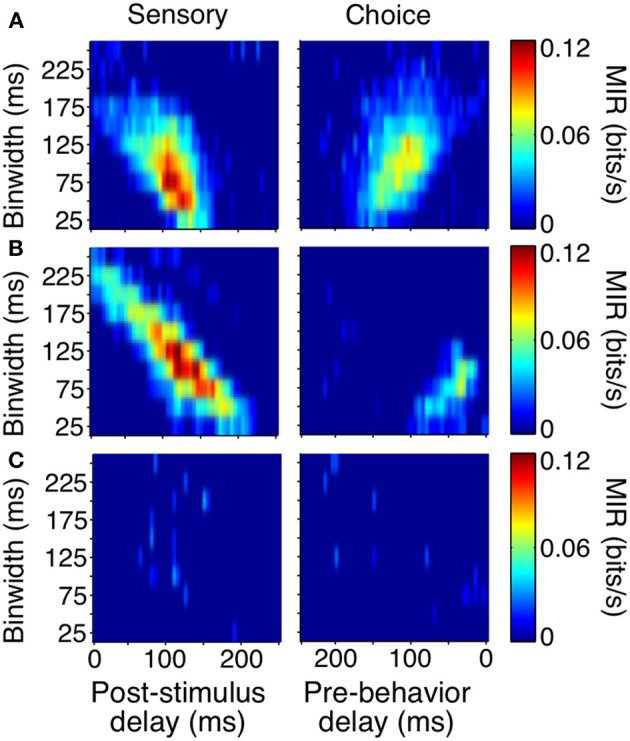
**Covariance- and bias-corrected sensory (left columns) and choice (right columns) surfaces corresponding to the example cells in Figure 3**. Color represents the magnitude of corrected MIR. The cells in **(A,B)** were some of the more reliable units about both the stimulus and the animal's behavior when specific bin separations and widths were used. The cell in **(C)** is representative of neurons lacking a well-defined peak on either surface. The diagonal appearance of the peak was to be expected if the neuronal response occurs with a consistent delay. As binwidth increased in steps of 25 ms, the neuronal response was considered 25 ms further into the trial.

As with the event-aligned average firing rates, information surfaces varied across individual neurons. Figure 6 shows the information surfaces corresponding to the example histograms in Figure 3. These surfaces indicated how the reliability (quantified as MIR) of each unit's relationship with the sensory stimulus and behavioral choice varied with the temporal parameters of the analysis window.

The cell in Figure 6A showed information peaks of similar magnitude on both the sensory and choice surfaces, while the cell in Figure 6B reflected the stimulus more strongly than the choice. However, for both of these neurons, moment-to-moment variations in firing rate over fine time scales (binwidths of 50–125 ms) significantly reflected the appearance of a shape and predicted the behavioral choice to either maintain fixation or make a saccade. Thus, at any point during the trial, observing the firing rate of one of these units over an ≈100 ms period would significantly improve one's chances at correctly determining if a shape had appeared ≈100 ms earlier (as reflected by the post-stimulus delay). Similarly, observing the firing rate of one of these units over an ≈100 ms period would improve one's chances, although to a slightly lesser extent, at correctly determining whether the animal would make a saccade in the next ≈125 or ≈50 ms (Figure 6A), as reflected by the pre-behavior delay. There were also units whose peak information rates were low and less well-defined (Figure 6C), indicating a lack of variations in firing rate that could be used to infer the stimulus or predict behavior on a moment-by-moment basis.

**Figure 7:**
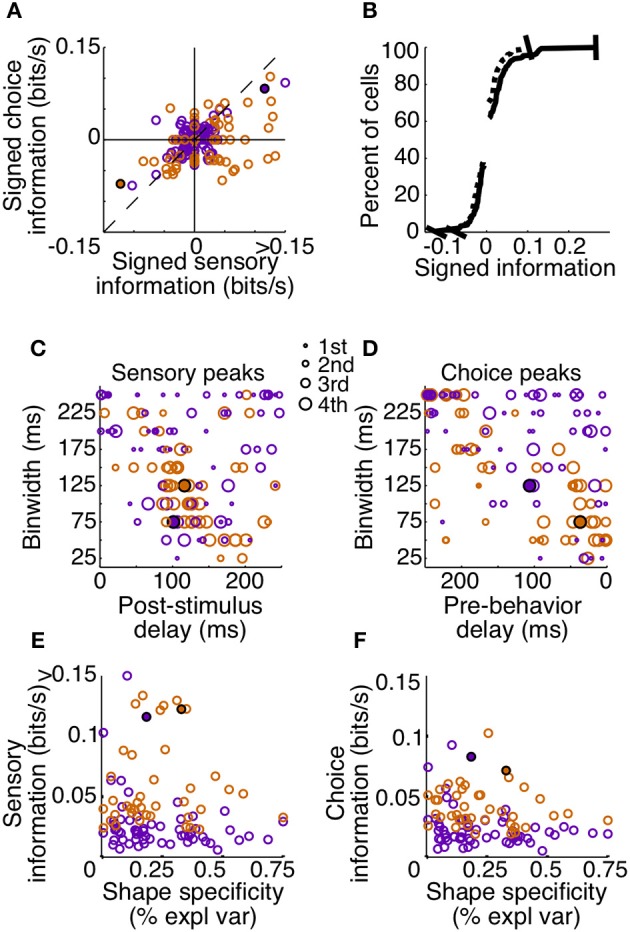
**Sensory and choice information for individual units**. Plots depict signed sensory information **(A,B)**, peak delay and resolutions **(C,D)**, and the relationship between reliability and shape specificity **(E,F)**. Points are colored by animal (Z: purple, J: orange), and filled circles indicate two of the example cells from Figures 3, 6. The third example cell lies at the origin in **(A)** and is absent from **(B–F)** because the number of significant points did not exceed the false alarm criteria on either surface. Many neurons in area V4 reflected both which stimulus was present, as well as the animal's subsequent behavior. Cells in quadrant 1 of **(A)** had significant sensory and choice modulation resulting from increased activity, while cells in quadrant 3 of **(A)** had significant sensory and choice information resulting from decreases in activity. The thin dotted line in **(A)** represents unity. In **(B–F)**, only units with significant sensory and choice information are shown (*N* = 99). Distributions from **(A)** were plotted cumulatively in (**B**, sensory: solid line, choice: dotted line). In **(C,D)**, circle size represents the quartile containing the unit's information, with the largest circles being the most informative units.

To summarize the reliability of all our sampled units, we relied on the peak, or maximum MIR, of the units' surfaces (Figure 7). We will refer to the maximum MIR of sensory and choice surfaces as a unit's sensory information and choice information, respectively. As evidenced by our example neurons, both positive and negative responses can reliably represent the stimulus state and be used to predict choices. While the quantity of MI can mathematically only be positive, at times it was helpful to compare the reliability of neurons with different directions of modulation. In these cases we plotted “signed information,” with the sign indicating whether the peak of a neuron's information surface was due to an increase or decrease in activity (Figure 7A). For example, negative-signed sensory information indicates that a cell's firing rate reliably decreased in the presence of the shape. To avoid issues of multiple comparisons within units, when representing an entire surface as a single point, we required that the number of significant points exceeded the false discovery rate based on the number of points considered. We also required the peak to be located between delays of 0–250 ms because larger delays would be non-causal to the animals' rapid detections. If these criteria were not met (as with the third example cell), the peak was considered to be zero.

Despite the fact that our stimulus was noisy and not optimized for the recorded units (other than receptive field location), a high percentage (133 out of 178, 75%) had significant sensory information with latencies shorter than the average reaction time. Thus, they would be potentially useful to the animal determining whether a shape was present. Of these, 74% (99 out of 133) also had significant choice information. The magnitudes of sensory and choice information are also comparable. If area V4 was reflecting only the stimulus, and was not related to the decision, the points in Figure 7A would all lie along the origin of the choice information axis; however, many points lie near the unity line. This suggests that in the context of the current task, area V4 may not only be representing the sensory stimulus, but its activity may directly influence decisions based on this representation.

Choice information could reflect a direct contribution of these units to the animal's behavior, or in a detection task such as ours, the choice information may reflect fluctuations in global factors (Nienborg et al., 2012). For example, if the animal is likely to detect the presence of a shape when he is closely attending the stimulus location, and this attention to this location causes an increase in firing rate of all units with receptive fields at that location, choice information may be more reflective of attentional locus than a contribution of individual neurons to the decision process.

The distribution of “signed” information can be used to put an upper bound on the contribution of global factors that modulate all units in the same way. In our data, if a given factor caused an increase in firing rate across a population, it would presumably increase the choice information of positively-modulated units but would decrease the information of negatively-modulated units. Differences in the predictive ability of units with negative choice modulations vs. positive choice modulations can thus be used to place an upper bound on the contribution of some global factors to choice information. For example, a previous study of motion detection and MT neurons found a subtle difference in choice propabilities between neurons that increased firing with the stimulus and neurons that decreased firing (Bosking and Maunsell, 2011). By contrast, using the choice information metric in our sample, the median choice reliability between positively and negatively modulated units is not statistically different (Mann–Whitney *U*-test, *p* = 0.83). Moreover, truly global modulations would create choice effects even in neurons with no sensory information, which, on average, we did not observe. Thus, the reliable relationships between the recorded activity and shape detection cannot be explained by global factors (but see Discussion regarding the potential of more selective effects).

The tendency of units with high sensory information to also have high choice information suggests that behavior might have been based on a selective weighting of more reliable V4 neurons. If these sensory responses were actually being used to guide behavior in a excitatory feedforward manner, we would expect choice modulation to occur in the same direction as sensory modulation. For example, if a cell is contributing to the animal's behavior and its activity decreases in the presence of a shape, the animal should also be more likely to report detection of a shape, regardless of the actual stimulus state, when this neuron is firing less. The majority of our observations are consistent with this relationship; in units with both significant sensory and choice information, 70% (69 out of 99) exhibit the same sign (quadrants 1 and 3, Figure 7A).

An important aspect of our task is that the fast and precise reaction times place temporal constraints on neuronal processing potentially underlying shape detection. If V4 units contribute in a causal feedforward manner to shape detection, the precision of the neuronal responses should be similar to that of behavior. Additionally, if stimulus evoked modulations caused behavior, the sum of sensory and choice peak delays should approximately sum to the peak behavior delay. Behavior was most reliable in binwidths of 100–150 ms with a delay of 200–275 ms (Figure 5B). Similarly, we found that individual units tended to be most reliable about the stimulus when binwidths of 50–150 ms were considered. The delay of these sensory peaks most often corresponded with bins whose front edge was separated from the stimulus by a delay of 75–150 ms with a binwidth of 50–125 ms (Figure 7C). The location of choice peaks was more diffuse (Figure 7D). Units with precision similar to that of behavior (peaks in smaller binwidths) tended to have peaks in bins whose latest edge was separate from the saccade by 0–100 ms. Choice peaks resulting from less precise responses were more likely to occur well before the behavior they were predicting.

### 3.3. Shape specificity and waveform duration

Our findings indicate that a relatively small proportion of neurons in V4 (Figure 7B) were highly informative about the appearance of shape and predictive of the animals' saccades. Given the known selectivity to contours in area V4, we wondered if these were particularly informative by virtue of their shape specificity. For example, a neuron with high sensory information might have either very strong responses to the appearance of a single type of shape or consistent responses across several or all shapes. Likewise, a neuron with high choice information might reflect shape-specific top-down influences, such as feature attention, which create a covariance between responses and behavior. If both sensory and choice reliability were strongly dependent on shape selectivity, the tendency for these two measures to be correlated might simply reflect differences in shape selectivity within our sample.

We used a two-factor analysis of variance to determine how strongly a cell's response to the appearance of a shape (Factor A) depended on the identity of that shape (Factor B). Shape specificity was quantified as the percent of explainable variance due to this interaction and was examined with respect to sensory and choice information (Figures 7E,F). We also calculated Spearman's partial correlation between shape specificity, sensory information, and choice information to quantify the strength of the pairwise relationships between these variables when accounting for correlation with the third variable. Neither the partial correlation between sensory information and shape specificity (*r* = 0.02, *p* = 0.82) nor between choice information and shape specificity (*r* = − 0.12, *p* = 0.22) was significant. Thus, selectivity does not seem to be a determinant in either the magnitude of sensory or choice information seen in individual units. However, the partial correlation between sensory information and choice information was significant and clearly dominant (*r* = 0.76, *p* < 0.001), indicating that the units that most reliably signaled the presence of the shape were also those with the strongest relationship to the animal's behavior, regardless of their shape specificity.

A previous study by Mitchell et al. (2007) used the duration of spike waveforms to separate V4 neurons into putative local interneuron and pyramidal classes and found that the effects of attention in V4 were greater in putative interneurons. Because relationships between neuronal responses and behavioral choice can reflect top-down effects (Nienborg and Cumming, 2009), we investigated whether putative interneurons in our sample displayed the highest choice information. We examined only single units (55 out of 178) and used the methods described in Mitchell et al. (2007). While our distribution appeared bimodal, it was not significantly so (Hartigan's dip test, *p* = 0.5), possibly because of the low number of single units. However, we found that 29% of our single units had spike durations less than 200 ms, extremely similar to the proportions found previously by Mitchell et al. (2007) (they found 43 out of 152 putative interneurons with durations less than 200 μs). We also applied a multi-dimensional waveform discrimination algorithm (Quiroga et al., 2004) to classify our cells into two classes, which produced similar numbers of putative interneurons. Neither of these methods suggested any significant relationship between putative neuron class and choice information. Similarly, there was no obvious relationship between putative neuron class and sensory information, the direction of sensory and choice modulation, or shape specificity.

### 3.4. Single unit predictions of behavioral dynamics

Because individual same-signed units carry both sensory and choice information over narrow epochs of time within the reaction time window, it is possible that the same brief changes in activity actually contributed to behavior. Sensory and choice surfaces essentially describe the probability relationships between neuronal discharge, sensory events, and choice events, respectively. This allows them to be combined multiplicatively to provide an estimate of the behavioral performance that could result solely from the unit under consideration. To predict behavior based on sensory and choice surfaces, we considered all possible sensory and choice delays that would sum to each behavioral delay and plotted the maximum MI product on the predicted behavioral surface before converting to MIR.

This method allows us to consider how the temporal properties of a neuron's responses constrain the contribution it might make toward shape detection. In particular, it allows us to exclude neurons with high sensory and choice information as contributing to the decision process if either the temporal dependence of their sensory or choice information was very inconsistent with behavior. For example, if behavioral information is maximal at a precision of 75 ms, and a particular neuron either carries no sensory or no choice information at that binwidth, then it cannot be significantly contributing to the decision process. Similarly, if behavioral information is maximal at a delay of 250 ms, and a particular neuron carries no sensory or choice information at consistent delays (sensory delay + choice delay = 250 ms), then it cannot be significantly contributing to the decision process.

**Figure 8:**
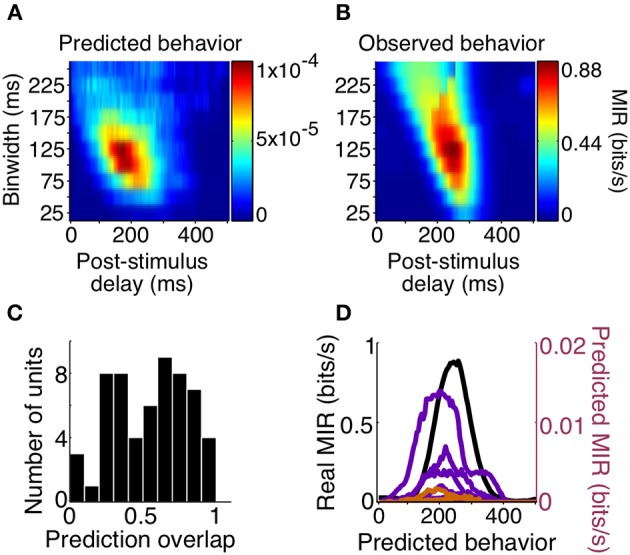
**Behavior predictions of same-sign units**. We combined sensory and choice surfaces of same-sign units (*N* = 69) to generate an average predicted behavior surface **(A)** which can be compared to the average of the real behavior surfaces (**B**, same as Figure 5B). A histogram of the overlap of the delay profiles of significant predicted (*N* = 58) and real behavior information at a binwidth of 125 ms is shown in **(C)**. The delay profiles of units with overlap >0.75 (*N* = 13, orange and purple, colored by animal) and the average of real behavior (black) are shown in **(D)**.

We found that the precision of the average predicted behavior surface very closely matched that of the observed behavior, with both peaks resulting from binwidths of 125 ms. However, predicted behavior MIR magnitudes were much lower than the animals' observed behavior, with the highest predicted behavioral reliability for any unit being approximately 100 times smaller than the observed reliability. Additionally, while delays corresponding to observed behavior were non-zero, the peak of the average predicted behavior surface occurred over delays that were too short (Figures 8A,B).

To determine how well the temporal parameters predicted by individual cells overlapped with those of the observed behavior we focused on how the MIR prediction changed with delay, using the most reliable binwidth for observed behavior (125 ms). We quantified the overlap as the normalized cross-product of the MIR prediction across delays at this binwidth. The median overlap was 0.58 (on a scale of 0–1), and there were 13 cells (7 positive signed information, 6 negative signed information) with overlap greater than 0.75. In this small population of cells, the periods over which the cells were informative about the stimulus, and informative about the animal's subsequent decisions, overlapped such that they could predict the timing of observed behavior reliability. The relationship between delay and MIR for these 13 units' behavior predictions, and for observed behavior, are all plotted in Figure 8. These results suggest that both increases and decreases in responses among a small number of the sampled V4 units are temporally consistent with a direct contribution to shape detection.

## 4. Discussion

We have shown that the activity of many V4 neurons is modulated by the brief presentation of contour shapes in a noisy background. This modulation often signals the presence of a shape, over timescales relevant to performance of the task, with significant reliability. Additionally, the responses of some of these same V4 neurons are also tightly linked to the behavioral report; prior to the report of a shape, regardless of whether a shape was actually present, the response of these neurons was altered. The direction and timing of task-relevant modulation in a few of these neurons suggests that a small percentage of neurons in area V4 may directly contribute to the rapid detection of shapes.

### 4.1. Sensory representation in V4 during rapid shape detection

We found that over short timescales, often tens of milliseconds, neurons in area V4 could signal, with significant reliability, the appearance of a shape through increases or decreases in activity, relative to the response of noise stimuli. Despite the fact that positioning the stimulus over the neurons' receptive fields was the only effort made at stimulus optimization, a large number of units (75%) conveyed some level of information about whether a shape was present. It is important to remember that our measure of sensory information is based on the task the animals were performing: the ability to indicate the appearance of any noise-embedded shape. Appropriately, the shape responses of some of the most reliable sampled units were only moderately selective for specific shapes, suggesting some degree of task-related invariance. These observations are consistent with the results of Chen et al. (2014). They showed that while the magnitude of responses to a collinear stimulus in area V4 depends on the orientation of the collinear elements, on average, individual cells were still able to signal the presence of the collinearity when it was rotated up to 60° away from the neurons' preferred orientations.

The observed sensory information could result from straightforward filtering of the shape and noise stimuli through V4 receptive field properties, resulting in both positive and negative response modulation (David et al., 2006). Feedback from areas in more anterior ventral visual, prefrontal, or parietal cortex or recurrent signaling with striate cortex could also all serve to enhance the activity of units representing a fragment of a shape, while suppressing the activity of units representing background/noise elements, provided such feedback were sufficiently fast and precise to be consistent with our observations of shape information precision. Functional MRI studies using very similar stimuli show increased responses to contours embedded in noise vs. noise only stimuli throughout early visual areas and the LOC (Altmann et al., 2003). While areas throughout the ventral visual stream are also likely to exhibit shape-appearance responses in the current study, recent work by Chen et al. (2014) showed that collinear stimuli embedded in noise modulated responses of single V4 neurons at the same latency as visual stimulus onset. This study also showed that contour-modulated responses in V4 actually preceded those of V1. Taken together, both of these observations suggest that purely feedforward processes maybe sufficient to create shape responses in V4 based on collinearity. Whether or not the shape representation originates in area V4, or is unique to the area, it is clear that within the window of the animal's reaction times, V4 neurons represent the appearance of shape embedded in noise with a precision similar to that of behavior.

### 4.2. Choice representation in V4 during rapid shape detection

The goal of this study was not only to establish whether V4 neurons were able to signal the sudden appearance of a shape within a noise stimulus, but also to determine if this information could directly contribute to behavior. Area V4 is reciprocally connected to prefrontal and parietal areas (Ungerleider et al., 2008; Ninomiya et al., 2012) that have been shown to accumulate evidence for decisions in sensory-based tasks (Shadlen and Newsome, 2001; Ding and Gold, 2012). In the context of extremely rapid visual decisions, it has also been suggested that V4 may directly initiate saccadic decisions (Kirchner and Thorpe, 2006). Area V4 is thus well-suited which to directly impact the visual-based decisions required by our task. We found that many of the V4 neurons with shape information were also statistically associated with the animal's moment-to-moment judgements of whether a shape was present, as indicated by significant choice information. In a subset of these units (*N* = 69), the firing rate modulations resulting in the highest sensory and choice information occurred in the same direction relative to the noise stimulus response. Most importantly, in a fraction of the same-direction units (*N* = 13), the sensory and choice modulations overlap in time so that they predict the behavioral delay between shape and response, as well as precision.

In discrimination tasks, correlations among and between neuronal pools tuned to the stimulus aspects to be discriminated may lead to non-causal relationships between a cell's response and the animal's behavior. In detection tasks such as ours, the main concern for non-causality is often that some global factor is correlated both with the animal's behavior and altered neuronal responses (Nienborg et al., 2012). One such factor is microsaccades, which have been shown to both affect the responses of V4 neurons (Leopold and Logothetis, 1998) and to explain at least some of the relationship between neuronal firing and behavior in other visual areas (Herrington et al., 2009). However, our results were very similar if analyses only included trials without microsaccades (data not shown).

Variations in top-down factors such as attention or arousal can serve as sources of covariance between neural activity and behavior (Cohen and Maunsell, 2011) and therefore contribute to choice correlations. However, such global factors are unlikely to be the sole source of our choice information observations. Because we only included units whose response increased significantly at the onset of the noise stimulus, at the time of shape appearance every unit included in this study was already responding to the noise stimulus with an increase in firing rate. This is true of cells both with zero sensory and/or choice information and with negative-signed information. A truly global factor would therefore induce choice information which varies little across the population. However, we observe a large variation in choice information across our sample. Moreover, if strong correlations were responsible for choice information, one should find positive choice informations even for neurons with no sensory information. By contrast, we do not observe such cells: similar to observations of putative motion detection signals in MT (Ghose and Harrison, 2009), significant choice information is almost exclusively found among cells with significant sensory information. Finally, in a study of motion signals within area MT, a deliberate modulation of attention did not create a universal effect on choice information across the population (Harrison et al., 2013).

Non-global attention effects, directed to specific populations, could potentially create choice information among certain neurons that was not reflective bottom-up contributions to detection. For example, David et al. (2008) showed that feature-based attention can alter the tuning of V4 neurons which could result in responses to particular stimuli being either enhanced or suppressed. However, in our study, there was no way for the animals to anticipate the particular shape that was going to be presented and there is no behavioral evidence of the animals having any shape biases. Thus, shape-specific attention seems unlikely to have contributed to choice information. However, it is also possible that feature attention was not directed to specific shapes but rather some attribute such as collinearity shared across the shapes. In this case, the neurons with the least shape specificity should have the strongest choice information, since feature attention variations would affect them across all shapes. However, we found no relationship between shape specificity and choice information (Figure 7). Thus, neither shape-specific nor shape-general feature attention is likely to have substantially contributed to our finding of significant choice information among select neurons.

Even though top-down motivational factors are unlikely to contribute to our measures of choice information, it is still possible that choice-related firing reflects a post-decision feedback signal of the impending saccade rather than a bottom-up contribution to the saccadic decision (Moore et al., 1998; Steinmetz and Moore, 2010). The distribution of delays at which choice peaks occurred did not allow for obvious division of peaks into distinct pre- and post-decision categories. Moreover, the duration of choice information in some individual cells suggests that a given period of choice-related activity may actually reflect the superposition of different processes. For example, our sample contains cells whose choice information and behavior prediction (e.g., the bimodal purple trace in Figure 8D) are consistent, by virtue of latency, with both a feedforward role in decision-making and a feedback role from the impending saccade. Thus, a single volley of activity might start out reflecting purely sensory events, transition to feedforward sensorimotor decision-making and in the end purely reflect the impending saccade (Platt, 2002). In this sense the distribution of choice information time courses may reflect proportionally different contributions of these processes to the responses of individual units. Units with peak choice reliability at very short delays likely have responses dominated by saccadic preparation. In these cells, there was likely a temporal gap between the activity resulting in sensory and choice information, leading to feedforward prediction of detection delays that were too short or a detection precision that was too coarse (Figure 8). This is similar to the finding of Ogawa and Komatsu (2006) that during a multidimensional search task, sensory representations and behaviorally relevant representations are segregated in time, with the former potentially reflecting feedforward inputs while the latter result from delayed feedback.

In other cells sampled in the present study, however, choice information became significant 100–200 ms before the behavioral event, and 13 cells were able to predict both the delay and precision of animal's detection decisions (with >0.75 temporal overlap). Given the ability of these cells to predict both the latency and precision of behavior, such cells may contribute directly to the formation of the foveation decisions. While the cell with the highest sensory reliability was about a fourth as good as the animals at detecting the appearance of shapes, the combination of sensory and choice surfaces predicted behavior orders of magnitude lower than the observed behavioral reliability. This suggests that many such cells may be required for the types of foveation decisions investigated here.

In such foveation decisions, sensory evidence regarding the presence of a shape must be propagated to oculomotor circuits. We have shown that the activity of area V4 neurons reflects this evidence and could be conveyed through well-established connections to oculomotor pathways. Furthermore, the activity of a few of these cells is both linked to the animals' choices and predicts temporal parameters of observed behavior. Therefore, we believe that the most parsimonious explanation for our data is that a small percentage of reliable V4 neurons contributed in a direct manner to rapid shape detection. It is also possible that shape detection decisions are based on a larger percentage of V4 neurons than indicated by studies of our sampled single cells. For example, some cells may contribute by coordinating their firing with other neurons, without changing their firing rate, or activity may be pooled over very large numbers of cells, such that neurons without measurable choice information in this data set actually do contribute to the decision. Such correlations could significantly impact the ability of a neuronal pool to explain detection reliability and precision in our task. Thus, an important avenue for further research is to investigate how pair-wise or even higher order correlations over small timescales affects the ability of neuronal populations to signal stimulus events and predict actions.

## Author contributions

Geoffrey M. Ghose and Katherine F. Weiner conceived and designed the experiment. Katherine F. Weiner performed the experiments and analyzed the data. Geoffrey M. Ghose and Katherine F. Weiner interpreted the results and wrote the paper.

## Funding

EY014989, NS076408, University of Minnesota Graduate School Fellowship, Frieda Martha Kunze Fellowship.

### Conflict of interest statement

The authors declare that the research was conducted in the absence of any commercial or financial relationships that could be construed as a potential conflict of interest.
